# UAV and UFB Detection Capability of an L-Band Long-Range Air Surveillance Radar: Geometric and RCS Constraints for LSS Targets

**DOI:** 10.3390/s26134180

**Published:** 2026-07-02

**Authors:** András Braun, Norbert Hegyi

**Affiliations:** 1 Doctoral School on Safety and Security Sciences, Óbuda University, H-1034 Budapest, Hungary; braun.andras92@stud.uni-obuda.hu; 2 Department of Vehicle Production and Engineering, Széchenyi István University, H-9026 Győr, Hungary

**Keywords:** unmanned aerial vehicle, unmanned free balloon, radar system, air surveillance, radar cross-section, radar detection range

## Abstract

The spread of unmanned aerial vehicles (UAVs) and unmanned free balloons (UFBs) has made ground-based air surveillance more difficult, especially for low, slow, and small (LSS) targets. Such targets often combine low radar cross-section (RCS), low altitude, small radial velocity, and strong coupling to ground clutter. This study provides a focused assessment of the detection constraints expected for a RAT-31DL-type long-range L-band surveillance radar against small UAVs and radiosonde-type light UFB payloads. The work combines simplified RCS estimates, literature-based UAV RCS data, finite element method (FEM) simulation, radar-horizon geometry, elevation-beam intersection analysis, and low-Doppler considerations. Idealized broadside reference RCS values are calculated at 1.5 GHz. Published 26–40 GHz UAV RCS data are used as comparison references and are back-scaled to the L-band to illustrate frequency-scaling uncertainty. A simplified FEM model of a trademark Meteomodem M20 radiosonde is simulated at 1.5 GHz and at 26 GHz for comparison, to examine aspect- and polarization-dependent scattering. The simulated radiosonde cross-polarized RCS values vary from approximately −36.49 to −23.45 dBsm at 1.5 GHz. For a 30 m radar installation and 60–140 m target altitudes, the smooth-Earth horizon-limited visibility range is approximately 55–71 km. Low-altitude coverage can be further limited by positive-elevation beam geometry. Taken together, the results indicate that LSS detectability is strongly scenario-dependent and is governed by RCS variability, geometric visibility, clutter, Doppler behavior, and radar-specific processing choices.

## 1. Introduction

Radar remains one of the basic tools of modern airspace surveillance and national security operations. Current systems have to deal not only with ballistic missiles and low-observable aircraft, but also with the growing use of unmanned aerial vehicles (UAVs). UAVs have changed both military and civilian aviation. From small commercial drones to tactical and strategic military platforms, they introduce new security concerns because of their small radar cross-section (RCS), low flight altitude, slow speed, and maneuverability [1,2,3].

Alongside powered UAVs, airspace surveillance systems also encounter unmanned free balloons (UFBs), i.e., lighter-than-air aircraft drifting with the wind. They usually carry meteorological or experimental payloads, but may also carry surveillance instrumentation such as radiosondes or reconnaissance payloads. From a radar perspective, UFBs matter because they can appear as very slow, weakly maneuvering targets with small radial velocity.

Hungary’s air defense infrastructure is integrated into NATO’s Integrated Air and Missile Defense System (NATINAMDS). Because of its Central European location, Hungary has an important role in maintaining a common regional air picture. This study places the RAT-31DL 3D radar (Leonardo S.p.A., Rome, Italy) in that surveillance context and examines its capability against small UAVs and UFBs through estimates of RCS, radar-horizon limits, and detection-range constraints.

The paper does not propose a new radar architecture, waveform, signal-processing algorithm, or theoretical detection method. Its purpose is more focused: to give a constraint-based engineering assessment of LSS detectability for a RAT-31DL-type L-band long-range air surveillance radar. The assessment focuses on uncertain L-band RCSs, radiosonde FEM results, radar-horizon geometry, elevation-beam intersection, low-Doppler vulnerability, and clutter effects. This framing separates the work from dedicated counter-UAS algorithm studies and treats the problem as a system-level detectability and deployment-planning question.

## 2. Basics of UAVs, UFBs, and Radars

### 2.1. Defining UAVs and UFBs

UAVs are aircraft that operate without onboard human personnel. They may be remotely piloted or may carry out parts of a mission autonomously using onboard systems. UAVs are generally grouped by mass, propulsion, endurance, airframe configuration, or autonomy level, and their mass ranges from a few grams to multi-ton aircraft. Using the maximum takeoff mass (MTOM) categories defined by the European Union Aviation Safety Agency (EASA), this study focuses on small UAVs below 4 kg, corresponding to C0, C1, and C2 class marks. These aircraft are typically operated up to 120 m above ground level (AGL) [4].

UFBs are lighter-than-air aircraft without onboard propulsion. They are also widely used in meteorology and scientific observations and can ascend into the upper atmosphere. Their payloads usually include radiosondes for in situ atmospheric measurements, sometimes combined with custom research, technology-demonstration, or educational instruments [5,6].

Within the European Union, the Commission Implementing Regulation (EU) No. 923/2012 sets out three UFB categories as light, medium, or heavy based on payload mass and related mechanical criteria [5].

Operations must be conducted in a manner that does not endanger people, property, or other aircraft. This is achieved through a combination of design provisions such as weak links and force-limited connections. In their most common configuration, a UFB includes three main elements: the balloon part, a parachute, and the payload [5,6].

From a materials perspective, balloons are typically manufactured from latex or polyethylene, selected for their strength, elasticity, and suitability for high-altitude expansion. UFBs usually reach altitudes of approximately 20–40 km. During descent, payloads are generally decelerated using a parachute. However, for specific mission profiles, a parachute-less descent may be acceptable when the payload mass, mechanical design, and operational constraints satisfy specified safety criteria [6].

Because UFB classification depends on several parameters, this study focuses on light unmanned free balloons (LUFBs), the category most often associated with radiosonde operations.

The basic classifying characteristics for LUFBs are:

- The payload suspension requires less than 230 N to separate the payload from the balloon;
- No individual payload package with an area density of 13 g/cm^2^ or more has a mass of 2 kg or more;
- No individual payload package with an area density of less than 13 g/cm^2^ has a mass of 4 kg or more;
- The combined payload mass is less than 4 kg [5].

**Figure 1:**
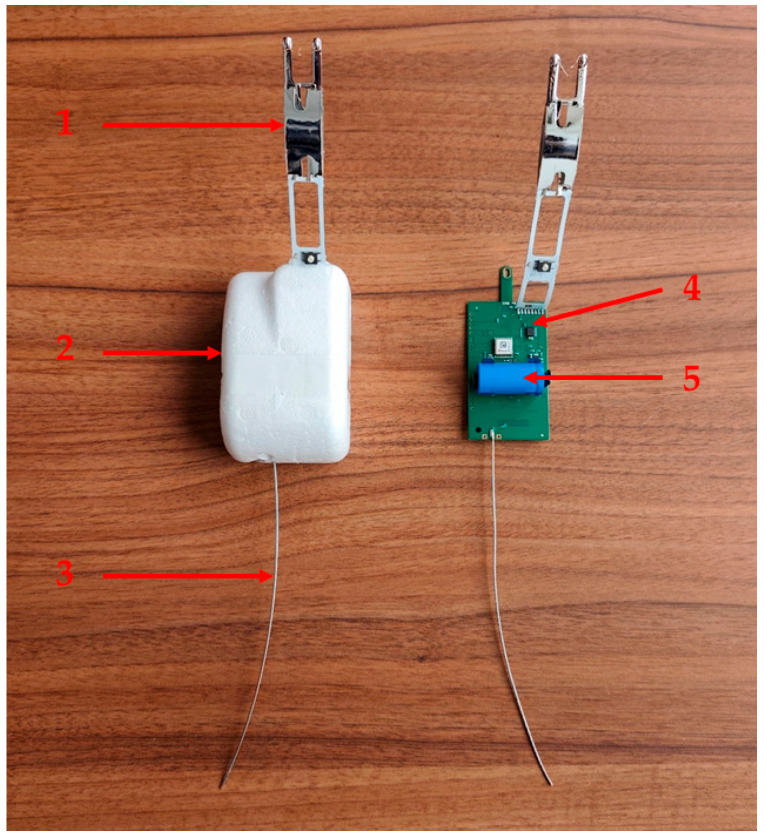
Meteomodem M20: sensor (1), body casing (2), antenna (3), PCB (4), battery (5).

Figure 1 gives an example of a commonly used radiosonde, the trademark Meteomodem M20 model (Meteomodem, Ury, France), showing both the outside and inside. From the outside, the radiosonde usually consists of an expanded polystyrene (EPS) body casing (denoted by no. 2 in Figure 1) with extending parts as sensors (denoted by no. 1 in Figure 1) or antennas (denoted by no. 3 in Figure 1). The interior comprises a printed circuit board (PCB) (denoted by no. 4 on Figure 1) and a battery (denoted by no. 5 on Figure 1) mounted to it.

The examined M20 unit has a mass of 36 g, and the main body measures 96 × 42 × 63 mm (height × length × width). The inner electronic component’s main size is 75 × 22 × 42 mm (height × length × width), including the PCB and mounted battery. The PCB (denoted by no. 4 in Figure 1), the CR123A-sized 3 V battery (denoted by no. 5 in Figure 1), the 0.5 × 132 × 16 mm outer meteorological sensors (denoted by no. 1 in Figure 1), and the 177 mm long, 1 mm diameter transmitting antenna (denoted by no. 3 in Figure 1), are shown.

### 2.2. Location and Properties of Radars

**Figure 2:**
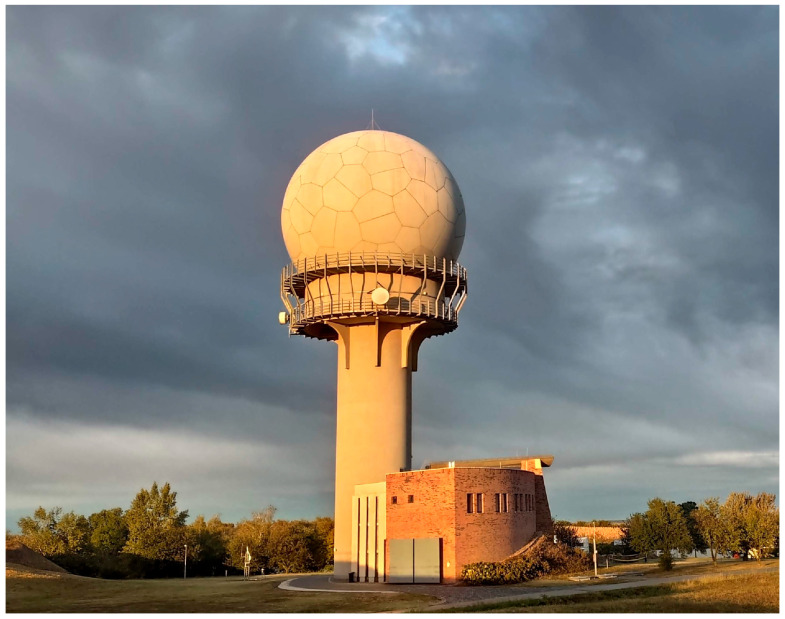
RAT-31DL 3D radar tower.

Five military radar sites currently operate in Hungary and form the backbone of national airspace defense. Three of them, near Bánkút, Békéscsaba, and Medina, use the RAT-31DL 3D radar, an Italian-made Leonardo system widely used within NATO. This radar uses Active Electronically Scanned Array (AESA) technology, with electronic beamforming and processing units able to track hundreds of targets at the same time [7]. Figure 2 shows the radar station at Medina.

The RAT-31DL operates in the L-band (1–2 GHz) and has a published instrumented range of 470 km. Track data are transmitted in real time to the Air Operation Command and Control Center, so detected threats in Hungarian airspace can be incorporated into the international air defense situational picture [8].

Table 1 lists the radar frequency bands and the corresponding wavelength ranges used in this paper.

### 2.3. Radar Detection Challenges for UAVs and UFBs

RAT-31DL-type radars are designed for long-range 3D air surveillance and are useful as strategic wide-area sensors in national and NATO-integrated air defense architectures. They should not be treated as replacements for short-range, purpose-built, counter-UAS (C-UAS) radars, whose waveforms, scan strategies, elevation coverage, and processing chains can be tailored to LSS targets [11]. In a layered system, the RAT-31DL generally provides the broad surveillance layer, while local C-UAS or multi-mission radars can improve coverage in demanding low-altitude regions.

Small UAV detection is different from conventional aircraft detection because these targets combine low RCS, low altitude, low speed, maneuverability, and strong coupling to ground clutter, interference, and biological movers [12].

Multirotor UAV signatures are useful only when Doppler resolution, signal-to-clutter separation, and classification methods are sufficient [13,14]. Larger tactical and strategic UAVs raise a different detection problem and are therefore outside the main scope of this LSS-focused assessment [15,16].

Radiosonde-type UFB payloads create a somewhat different detection problem. Their motion is driven mainly by wind drift, they normally have no rotor-induced micro-Doppler, and their echoes may remain close to the zero-Doppler region, where clutter-suppression processing can weaken slow-target returns. For radiosonde-type UFBs, radar-horizon limitations matter most during launch, early ascent, descent, and landing. At high altitude, geometry is less restrictive, while RCS, aspect angle, polarization, and tracker behavior become more important.

**Figure 3:**
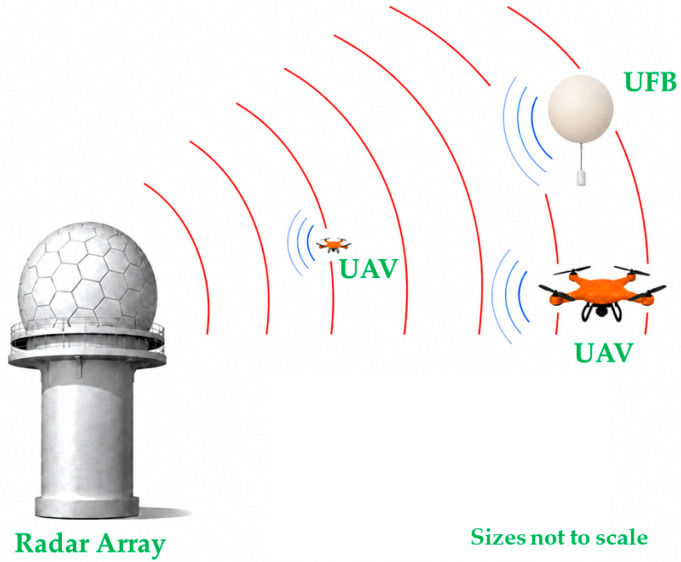
Visualization of UAV and UFB detection by radar station.

Larger tactical or strategic UAVs should be considered separately because their RCS and flight profiles can differ substantially from C0–C2 small UAVs. Their detection may also depend on range, autonomy, payload configuration, and possible low-observable design features [17,18]. The present study therefore maintains its focus on the LSS case, where small size, low altitude, low Doppler, clutter coupling, and aspect-dependent RCS are the main limiting factors. Figure 3 illustrates the possible radar detection of UAV and UFB targets.

RAT-31DL-type long-range surveillance radars can generally support the detection of larger UAVs and geometrically visible UFBs when RCS, clutter, beam geometry, and processing conditions are favorable. Small UAVs and radiosonde-type UFBs, however, should be treated as distinct LSS subclasses. Small UAV detection is mainly limited by low altitude, clutter coupling, small RCS, aspect variability, and the possible need to exploit micro-Doppler signatures. UFB detection is also affected by near-zero-Doppler behavior and weak classification indicators. These differences, in practical terms, support a layered surveillance concept: RAT-31DL supplies strategic wide-area coverage, while local sensors fill low-altitude gaps and support classification and confirmation. Table 2 summarizes these differences using LSS air threat literature, radar micro-Doppler studies, official UFB regulation, meteorological balloon observation guidance, and C-UAS sensor fusion references [19].

## 3. RCS, Radar Horizon, and Filtering Effects

### 3.1. Calculations for Possible RCSs

For C0, C1, and C2 UAVs, most dedicated counter-UAV radars use higher carrier frequencies than the L-band, typically the X-band (8–12 GHz) or the Ku/K/Ka/W bands (12–110 GHz). Shorter wavelengths generally provide better angular resolution, target discrimination, and small-target detectability. This study, however, focuses on the IEEE-defined L-band (1–2 GHz) [20,21,22].

Counter-UAS sensing reviews show that many purpose-built UAV detection radars operate in the X-band and higher bands, including the K- and W-band. Frequencies above about 6 GHz are usually better suited to small, slow, low-flying targets. Lower-band radars, however, may be useful in selected architectures; for example, specialized multibeam staring radars. Measurements around 1.5 GHz also show that small-UAV returns depend strongly on scenario and can be confused with birds or other non-UAV movers, which makes geometry- and clutter-aware processing important at lower frequencies [20,23].

Idealized Perfect Electric Conductor (PEC) reference shapes provide a more focused first estimate of how small the RCS of UAVs and LUFB payloads may be. In this study, the RCS is reported both in square meters and in decibels relative to one square meter (dBsm). The dBsm scale is useful because radar targets span a large dynamic range and because link-budget terms are commonly handled in decibels. It also provides a compact way to compare how the RCS changes with frequency, aspect angle, polarization, and target type.

The estimates assume a free-space target in air, treated as electromagnetically isolated and not intentionally grounded. RCS values are calculated for ideal orientation: broadside, normal incidence for planar faces, and broadside presentation for cylindrical cells. The geometry is deliberately simplified, with EPS, XPS, and PCB assemblies represented as smooth planar faces.

The monostatic RCS is calculated as [24]:

(1) $$\sigma =\frac{4\pi A^{2}}{\lambda^{2}}.$$

The RCS in dBsm is given by [24]:

(2) $$\sigma_{dBsm}=10\cdot \log_{10}\left(\sigma \right),$$

where σ is the RCS, A is the area, and λ is the wavelength.

The wavelength is [24]:

(3) $$\lambda =\frac{c}{f},$$

where f is the frequency and c is the speed of light.

The wavelength is calculated from the chosen frequency:

f = 1.5 GHz;

c = 299,792,458 m/s;

λ = 0.19986 m.

Real UAVs and radiosondes are not flat PEC plates, and off-broadside aspects usually reduce RCS substantially. The PEC broadside plate should therefore be read as an idealized reference case, not as a strict physical upper bound.

Commercial micro-UAVs span a wide size range. Representative platforms (e.g., DJI RoboMasterTT) have characteristic dimensions on the order of 100 mm, with central fuselage dimensions about 40 mm [25].

As noted in Section 2.1, radiosondes such as the Meteomodem M20 can be even smaller than many commercial micro-UAVs used for comparison. In practice, aspect angle, curvature, and material losses generally reduce the RCS, and the value can change substantially with orientation.

The material estimates in this section are interpreted mainly for the 1.5 GHz L-band case. The additional 26 GHz values are included only as additional references for comparison with published mm wave UAV RCS data.

At the L-band, the examined square facets are electrically small to intermediate. At 1.5 GHz, for example, 50 × 50 mm and 100 × 100 mm plates correspond to approximately 0.25–0.50 wavelengths. In this range, a simple broadside specular plate model is not fully reliable. Induced currents are not uniform, and the return may depend strongly on edge and corner diffraction, aspect angle, polarization, and resonant current modes. For this reason, the broadside PEC plate values are used only as idealized reference estimates, not as operational RCS predictions [26,27,28].

As discussed in Section 2.1, the main parts of a modern radiosonde are usually EPS, FR-4 PCB laminate, and batteries. Metallic battery cans, such as CR123A-size cells, can dominate backscatter but are excluded from this first-order material-only estimate. Dry EPS generally has permittivity close to air, so a smooth EPS surface reflects very little at 1.5 GHz and most energy passes through it unless the block is large or contains embedded conductors. If the foam becomes wet, its effective permittivity and loss increase, and the surface can become more reflective. FR-4 is a higher-permittivity dielectric, so a bare FR-4 face reflects more than foam. In real PCBs, however, returns are often dominated by copper planes, traces, shielding, and component leads, making the response closer to that of a conductive reflector. Additional calculations were therefore made to estimate possible upper-range radiosonde RCS values.

For a non-magnetic dielectric at normal incidence, the reflection coefficient and the power reflectivity are given by [29]:

(4) $$\Gamma =\left(\frac{\sqrt{\varepsilon_{r}}-1}{\sqrt{\varepsilon_{r}}+1}\right),$$

(5) $${\left|\Gamma \right|}^{2}={\left(\frac{\sqrt{\varepsilon_{r}}-1}{\sqrt{\varepsilon_{r}}+1}\right)}^{2}.$$

To provide best-case, order-of-magnitude monostatic RCS values, idealized geometries are used.

Dielectric-scaled approximation equation (first-order upper bound) [29]:

(6) $$\sigma_{dielectric}=\sigma_{PEC}\cdot |\Gamma {|}^{2}.$$

These expressions are used as a simple reference framework. The PEC plate gives the broadside specular case, dielectrics are scaled using normal-incidence power reflectivity, and the PCB is represented by an effective coherent conductive area.

Table 3 gives idealized broadside, smooth-face, monostatic RCS reference values. EPS and FR-4 values are obtained by scaling a PEC plate RCS with the normal-incidence power reflectivity |Γ|^2^. The reference geometries are the previously used 50 × 50 mm and 100 × 100 mm square plates. These parameters are intended to approximate an idealized upper range for the RCS.

The material calculations in Table 3 give very low RCS values for the radiosonde-related materials. Dry EPS produces almost no backscatter at 1.5 GHz, while PEC and FR-4 provide broadside reference limits rather than realistic target signatures. The PEC case is an optimistic broadside reference, and real PCBs may exceed the simple FR-4 estimate because of copper traces, ground planes, batteries, antennas, and other conductive components. The dielectric-scaled and PEC flat-plate values should therefore be interpreted only as order-of-magnitude material-contrast indicators. Actual UAVs and radiosonde RCSs may, however, differ substantially because of geometry, conductive parts, moisture, resonances, aspect angle, and polarization.

**Figure 4:**
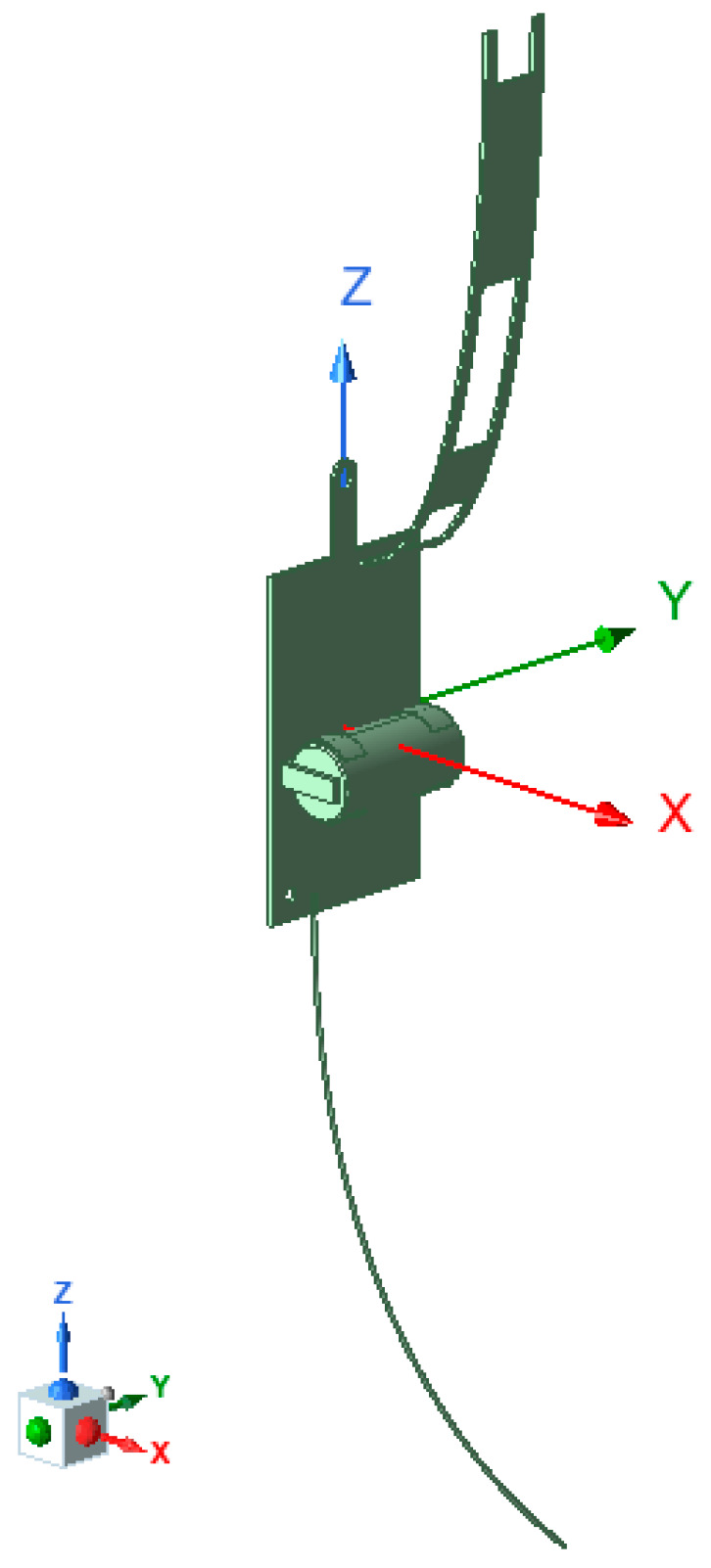
Simplified 3D model from FEM simulation.

For the FEM analysis, the RCS of the M20 radiosonde was simulated at 1.5 GHz for multiple aspect angles using Ansys Electromagnetics 2025 R2 (Ansys, Inc., Canonsburg, PA, USA). The analysis also used a simplified electromagnetic model of the radiosonde, shown in Figure 4.

The FEM model excludes the dry EPS casing because its low dielectric contrast at the L-band should scatter much less than the conductive elements. The model therefore concentrates on the PCB metallization, sensors, antenna, and small metallic parts. As stated previously wet, icy, or moisture-contaminated EPS may change the scattering response. However, returns are often dominated by metallic components, making the response closer to a conductive reflector. This should be examined in later sensitivity studies.

Table 4 lists the nominal material parameters assigned to the conductive components in the simplified FEM model. Copper is used for PCB metallization and sensors, while stainless steel is used for the antenna and small metallic parts. The conductivity values are representative engineering inputs, and the corresponding skin depths at 1.5 GHz and 26 GHz were calculated in Ansys Electromagnetics. Thus, Table 4 should be read as a modeling-parameter table, not as certified material measurements of the specific radiosonde sample.

**Figure 5:**
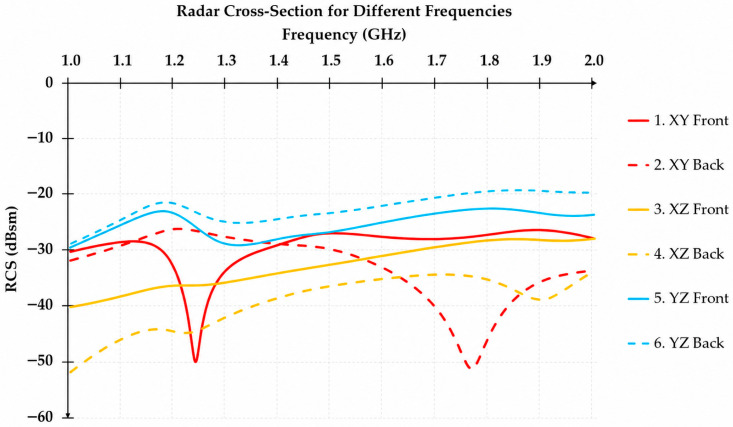
Simulated values for six representative aspect directions for cross-polarized RCSs: XY Front (1); XY Back (2); XZ Front (3); XZ Back (4); YZ Front (5); YZ Back (6).

Small PCB-mounted components were omitted because their size was not expected to affect the L-band response. The FEM model was solved in Ansys Electromagnetics 2025 R2 using incident plane-wave excitation, Cartesian polarization, and a 1–2 GHz sweep in 10 MHz steps. For conductive parts, skin-depth-based refinement used the values in Table 4, since surface currents dominate their scattering at 1.5 and 26 GHz. Mesh convergence was checked by repeating the 1.5 GHz and 26 GHz solution with refined meshes and comparing selected monostatic cross-polarized RCS values. The mesh was accepted when changes stayed below 1 dB. Figure 5 shows six representative aspect directions.

For further, more accurate analyses the 1.5 GHz results were extracted for discussion, presented in Table 5.

To support the later comparison with published 26–40 GHz UAV RCS measurements, the simplified M20 radiosonde FEM model was also evaluated at 26 GHz. This additional simulation is not part of the RAT-31DL L-band assessment; it provides only a common-frequency reference for comparing radiosonde-like LUFB and UAV scattering levels. The extracted 26 GHz M20 values are presented in Table 6.

Since the radar is sensitive to cross-polarized returns, the FEM results are relevant for interpreting the radiosonde response. At the same time, the values show that this response can change strongly with target orientation. Therefore, the tabulated RCS values should be read as cross-polarized reference cases, not as one fixed RCS value for the payload.

Estimates for drones can also be calculated by using RCS signatures of drone models measured between 26 GHz and 40 GHz frequencies. Using the reported measurements of Semkin et al. (2020) [33], the mean RCS values are presented in Table 7.

The cross-polar channels are clearly lower and more irregular e.g., DJI Matrice 100 (SZ DJI Technology Co., Ltd., Shenzhen, China), consistent with strong polarization sensitivity and shifting scattering contributions across frequencies. These oscillations show that the mean return is frequency-selective and strongly affected by polarization and complex airframe scattering [33].

To provide an approximate L-band magnitude for illustration only, the 26–40 GHz RCS values can be back-scaled to 1.5 GHz. Conversion is presented for both sigma proportional to f^2^ and sigma proportional to f^4^. In dB form, the generalized conversion is presented in Equation (7) [24].

(7) $$\sigma_{dBsm}\left(f_{2}\right)=\sigma_{dBsm}\left(f_{1}\right)+10n\log_{10}\left(\frac{f_{2}}{f_{1}}\right),$$

where f_1_ is the original frequency (e.g., 26 GHz), f_2_ = 1.5 GHz, *n* = 2 (represents the first-order specular-like assumption), and *n* = 4 (represents an electrically small or Rayleigh-type sensitivity case).

With this model, the band-averaged converted mean estimates are summarized in Table 8.

The f^4^ case lowers the estimated 1.5 GHz RCS by an additional 24.8–28.5 dB relative to the f^2^ case. This shows that frequency extrapolation is one of the dominant uncertainty sources in the UAV RCS assessment. Table 8 summarizes these RCS estimates. These converted values are illustrative only. Real UAVs contain mixed materials, and their dominant L-band scattering mechanisms may differ from those measured at 26–40 GHz.

### 3.2. Calculation for Possible Radar Horizon

One of the more important physical limitations of low-altitude radar detection is the radar horizon, which is determined by Earth curvature, radar antenna height, target altitude, and atmospheric refraction. For UAVs and radiosonde-type UFB payloads below a few hundred meters, radar horizon can dominate detection range regardless of transmitter power or receiver sensitivity.

In practical terms, this means that increasing radar sensitivity cannot compensate for a target that is still below the local line of sight. For LSS targets, the first question is therefore not only whether the echo is strong enough, but whether the object is geometrically exposed to the antenna at all. This is why the radar-horizon calculation is treated here as a separate limiting condition before the later detectability assessment.

UFBs can rise to 20–40 km, where horizon limits are weaker, but their launch, early ascent, descent, and landing remain affected by terrain, clutter, and low radial velocity.

This section evaluates the height–range relationship for a RAT-31DL-type L-band long-range 3D surveillance radar using the standard 4/3 Earth-radius model. The analysis focuses on low-altitude targets and separates two related but different constraints: smooth-Earth radar-horizon visibility and positive-elevation beam-axis intersection.

The radar horizon distance is approximated by equation [34]:

(8) $$d_{km}=\frac{\sqrt{2kR_{E}}}{1000}\left(\sqrt{h_{r}}+\sqrt{h_{c}}\right),$$

where *d_km_* is the radar horizon distance (km), *h_r_* is the radar antenna elevation AGL (m)*, h_c_* is the target altitude AGL (m), *R_E_* is the Earth radius (m), and *k* is the effective Earth radius factor.

The beam-target geometric intersection distance formula by equation [34]:

(9) $$d_{\theta ,km}=\frac{h_{c}-h_{r}}{1000\cdot tan(\theta )},$$

where *d_θ, km_* is the last beam-target intersection distance (km), *h_c_* is the target altitude AGL (m), *h_r_* is the radar antenna elevation AGL (m), and *θ* is the elevation tilt angle (°).

The calculations assume a 30 m radar antenna height and target altitudes from 60 m to 140 m AGL in 20 m steps. This altitude range generally represents typical low-altitude UAV- and UFB-relevant cases near the small civil-UAV operating ceiling. Very low flights are excluded because local terrain and clutter would dominate practical detection. Table 9 reports the radar-horizon and beam-axis intersection ranges calculated with Equations (8) and (9).

This choice also keeps the calculation close to the altitude band where geometric effects are most critical. At these heights, even a small change in target altitude or beam elevation can noticeably shift the available range. The values therefore serve mainly to show the sensitivity of low-altitude coverage, rather than to define a fixed operational limit.

For the positive-elevation cases, the values in Table 9 are not full detection envelopes. They represent only simplified beam-axis intersection ranges. Real coverage also depends on finite beamwidth, beam scheduling, sidelobes, terrain masking, clutter, detection thresholds, tracking logic, and integration gain.

Elevation angles from 2.5° to 20° are used as representative positive-elevation examples rather than confirmed operational RAT-31DL beam positions. This approach is therefore used to show how rapidly low-altitude beam-axis coverage decreases as elevation angle increases.

**Figure 6:**
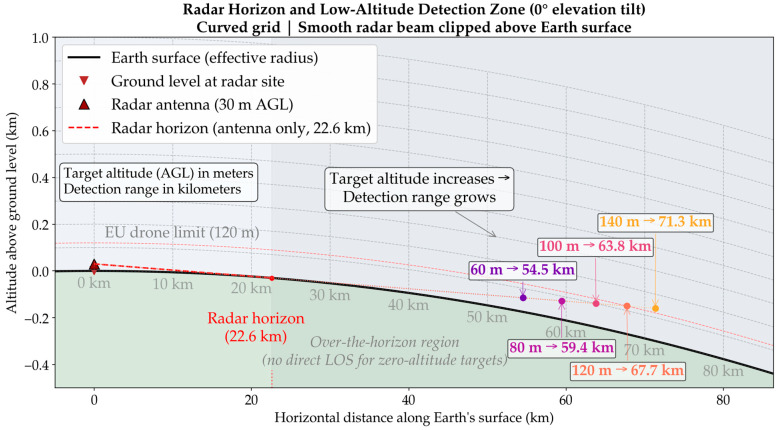
Visualization of 0° tilted array’s possible radar horizon with target altitudes at 60 m, 80 m, 100 m, 120 m, and 140 m AGL.

The results show that the 0° radar-horizon range increases with the square root of target altitude rather than linearly. Increasing the target altitude from 60 m to 140 m extends the theoretical smooth-Earth horizon from approximately 54.5 km to 71.3 km, or by about 16 km. However, even modest positive elevation angles sharply reduce the beam-axis intersection range for low-altitude targets. At the highest examined elevation angle, the beam-axis intersection range becomes extremely short for low-altitude targets. For example, at 20° elevation and 60 m target altitude, the calculated beam-axis intersection distance is only about 80 m. This shows that the main-beam axis reaches the 60 m altitude almost immediately after leaving the radar site. Consequently, such high positive elevation angles are not suitable for maintaining low-altitude beam-axis coverage at operationally relevant ranges in this simplified geometry. This confirms that low-altitude coverage is constrained not only by radar horizon, but also by elevation-beam geometry, as visualized in Figure 6.

**Figure 7:**
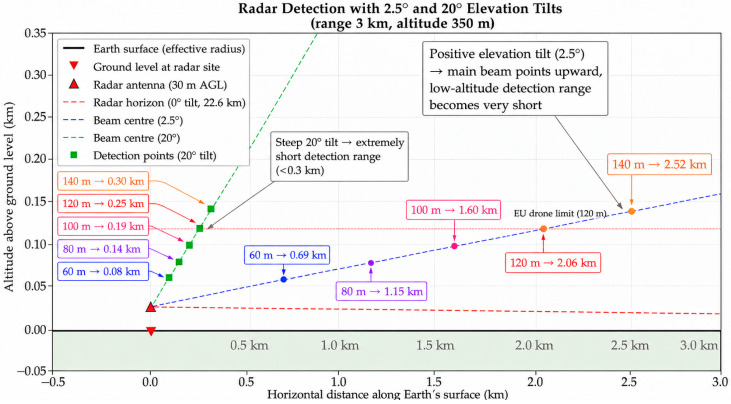
Beam-axis intersection geometry for the 2.5° and 20° positive-elevation boundary cases, visualizing for target altitudes at 60 m, 80 m, 100 m, 120 m, and 140 m AGL.

The second, more important part of the calculations focused on the effect of the elevation tilt of the antenna. The RAT-31DL radar uses electronically controlled beamforming in radar elevation, but the direction of each beam determines how far the axis of the main beam intersects the given target altitude. At positive elevation tilt, the main beam rises upwards, so it can only cover low-flying targets for a limited distance. Figure 7 visualizes the lower and upper positive-elevation boundary cases considered in Table 9.

The analysis shows that advanced electronic beam steering and signal processing cannot remove fundamental geometric visibility limits. In practice, a target is detectable only if the beam clearance exceeds local terrain, obstacles, and a sufficient safety margin. Terrain masking, forest cover, buildings, towers, hills, near-ground multipath fading, and unusual refraction may therefore reduce or distort the theoretical smooth-Earth coverage.

### 3.3. Parametric Radar-Equation Detectability Assessment

Because detailed RAT-31DL waveform, receiver, integration, loss, CFAR-threshold, and processing-gain data are not public, this section uses a parametric radar-equation analysis rather than proprietary performance claims. The analysis separates three constraints: thermal-noise-limited detection, clutter-limited detection, and geometry-limited visibility.

For a monostatic radar, the signal-to-noise ratio can be expressed as [35]:

(10) $$SNR=\frac{P_{t}G_{t}G_{r}\lambda^{2}\sigma G_{i}}{{\left(4\pi \right)}^{3}R^{4}k_{B}T_{0}BF_{n}L},$$

where P_t_ is the transmitted power, G_t_ is the transmit antenna gain, G_r_ is the receive antenna gain, λ is the radar wavelength, σ is the radar cross-section of the target, G_i_ is the integration gain or processing gain, R is the radar-to-target range, k_B_ is the Boltzmann constant, T_0_ is the reference noise temperature, B is the receiver bandwidth, F_n_ is the receiver noise figure expressed as a linear factor, and L is the total system loss factor.

Equation (10) is used only to derive the normalized RCS sensitivity in Table 10 because the data needed for absolute RAT-31DL SNR, detection range, and probability-of-detection calculations are not public. The resulting values are therefore relative engineering estimates, not operational performance predictions.

With all other parameters fixed, the maximum thermal-noise-limited detection range scales as

(11) $$R_{max}\propto \sigma^{1/4}.$$

The relative range factor in Table 10 is calculated using a 0 dBsm reference target. Thus, 0 dBsm corresponds to 1 m^2^, and each dBsm value is first converted to a linear RCS before applying the fourth-root range law:

(12) $$0\;dBsm\;=\;1\;m^{2};$$

(13) $$\sigma =10^{\frac{\sigma_{dBsm}}{10}};$$

(14) $$R_{rel}=10^{\frac{\sigma_{dBsm}}{40}}.$$

Therefore, the relative range factor expresses the fraction of the 0 dBsm target range obtained under otherwise identical radar-equation parameters. This fourth-root dependence is central to engineering interpretation.

Consequently, a long-instrumented range cannot be directly converted into reliable LSS target coverage. Table 10 shows the detection-range sensitivity in connection with small-target RCSs.

### 3.4. Software Filtering

Signal processing is central to low-altitude LSS detection because slow UAVs and UFB payloads can fall close to the zero-Doppler clutter region. In this regime, the target return is weak because of small RCSs and is also hard to separate from stationary or slowly varying background returns. Clutter suppression, Doppler filtering, CFAR thresholding, and tracker logic therefore influence whether a low-altitude LSS target is retained or rejected after initial detection.

Moving Target Indication (MTI) suppresses stationary or near-stationary returns and preserves targets with sufficient Doppler shift [35,36]. This is effective against fixed ground clutter, but it can create a vulnerability for targets with very low radial velocity. A hovering UAV, a UAV moving tangentially to the radar line of sight, or a wind-drifted radiosonde payload may remain close to the clutter notch. If the processing chain is tuned mainly for conventional aircraft and strong clutter rejection, such targets may be weakened before detection or may fail to form a stable track.

The Doppler shift of a monostatic radar target is given by equation [24,35]:

(15) $$f_{D}=\frac{2v_{r}}{\lambda },$$

where v_r_ is the radial velocity, and λ is the radar wavelength.

At 1.5 GHz, λ is 0.2 m. Consequently, even moderate physical motion can produce only a small Doppler shift when the radial component is weak. Table 11 illustrates this sensitivity and shows why UFBs and tangentially moving UAVs can occupy the same Doppler region as residual ground clutter.

Table 11 shows that radiosonde-type LUFB payloads and tangentially moving or hovering UAVs can remain in the low-Doppler region when their radial velocity component is small. These values should not, however, be read as universal rejection thresholds or as proof that such targets are automatically undetectable.

RAT-31DL-type L-band surveillance radars are designed mainly for conventional aircraft, including fast jet targets with much larger Doppler shifts; low Doppler alone therefore does not rule out detection. For UAVs and radiosonde-type LUFB payloads, however, low Doppler can also reduce robustness when it coincides with a small RCS, low altitude, clutter coupling, and radar-specific filtering or tracker settings. Because RAT-31DL processing parameters are not public, this analysis does not assign a fixed Doppler rejection threshold. Whether slow components are preserved, reduced, or rejected depends on the radar’s PRF, coherent processing interval, Doppler-filter bank, MTI/AMTI notch characteristics, clutter-map configuration, local clutter environment, and tracker logic. Adaptive Moving Target Indication (AMTI) extends the MTI principle by using adaptive filtering and motion compensation to suppress broadened clutter spectra [35,36].

Although AMTI is usually discussed in connection with moving-platform radars, the same engineering trade-off appears here. Stronger clutter rejection reduces false alarms, but it can also reduce sensitivity to slow, low RCS targets. This is especially relevant for UFBs, which drift with the wind, and for UAVs whose radial velocity can be small during hovering, orbiting, or cross-range motion.

Constant False Alarm Rate (CFAR) processing adjusts the detection threshold to local noise and clutter. CA-CFAR works well in homogeneous clutter, while OS-CFAR, GO-CFAR, and SO-CFAR are generally more robust near clutter edges or in heterogeneous clutter. In low-altitude surveillance, however, reference cells may contain terrain, vegetation, discrete scatterers, birds, weather returns, or moving vehicles. In such cases, the adaptive threshold can rise and mask weak UAV or UFB echoes [35,37].

Low-Doppler-preserving processing, such as low-Doppler maps or zero/near-zero-Doppler channels, can help by retaining returns that conventional clutter-rejection filters might suppress [35,37]. Preserving these channels can improve detection of slow LSS targets, but it can also increase false alarms from residual clutter, birds, vegetation, precipitation, and other low-Doppler background sources. The processing configuration must therefore balance detection sensitivity against false alarm control, rather than simply maximize clutter suppression.

At L-band, low-altitude LSS detection is often limited by clutter rather than by receiver noise alone. Relevant clutter sources include terrain, vegetation, buildings, towers, wind turbines, vehicles, precipitation, and biological movers [12,20,23]. Bird clutter is particularly important because birds can occupy altitude, speed, and RCS regimes similar to small UAVs. Multirotor UAVs may be separated by blade-related micro-Doppler components, whereas UFBs normally lack propulsion-induced micro-Doppler and may remain close to zero Doppler. Reliable classification should therefore combine RCS, Doppler, micro-Doppler, trajectory, persistence, and contextual information.

For RAT-31DL-type long-range surveillance radars, public sources describe the general L-band surveillance role but not the detailed MTI, AMTI, CFAR, clutter-map, or tracker parameters [38]. The discussion above should therefore be read as a physically motivated processing-sensitivity analysis, not as a statement about a specific proprietary radar mode. The operational point remains important. An LSS target may be geometrically visible; however, it may remain difficult to detect or track if it falls into a suppressed Doppler region or if local clutter raises the adaptive detection threshold.

Sensor fusion can reduce these limitations by combining optical, infrared, acoustic, passive RF, mobile radar, and multistatic or networked radar data. These complementary views improve resilience when one sensor is shadowed, clutter-limited, or affected by poor data quality [20]. In this sense, software filtering should not be treated in isolation. It is one part of a layered LSS surveillance architecture that has to coordinate radar detection, Doppler processing, classification, tracking, and multisensor correlation.

## 4. Results and Discussion

This study provides a quantitative, geometry-based assessment of low-altitude target-detection limitations for a RAT-31DL ground-based, long-range, L-band 3D air surveillance radar. The analysis focuses on radar-horizon geometry and antenna-elevation beam geometry, using simplified but physically consistent models intended to reflect realistic operating constraints.

### 4.1. Expected RCS for UAV and UFB Targets

At the selected L-band operating point, typical micro-UAVs (C0–C2) and UFBs, particularly radiosonde-sized objects, are electrically small or only marginally sized. Their expected radar returns are therefore low and highly sensitive to aspect angle, material composition, and the presence or absence of specular-like surfaces. The calculations are intended to give order-of-magnitude insight into the small RCS character of these targets, not definitive σ values.

Equations (1) and (2) were applied to square reflectors viewed broadside, representing an idealized broadside reference case summarized in Table 3. A clear scaling emerges: increasing the square side length from 50 mm to 100 mm increases σ from −27.06 dBsm to −15.02 dBsm. For this ideal broadside plate case, the trend is consistent with σ ∝ A^2^, meaning that even modest increases in projected area can produce disproportionately stronger returns. However, real UAVs and radiosonde-sized UFB targets are not ideal plates, and off-broadside aspects typically reduce σ substantially below these optimistic values.

These values are not strict upper bounds. In the electrically small-to-intermediate regime, diffraction, resonance, polarization, and aspect angle can produce strong peaks and nulls.

In practice, curvature, seams, apertures, and material losses (e.g., plastics and composites) redistribute scattering and reduce effective RCSs. Additional time variability is introduced by orientation changes and maneuvering, rotor-induced micro-motions, and associated fluctuations in aspect angle and dominant scattering centers.

The FEM simulation of the simplified Meteomodem M20 radiosonde at 1.5 GHz supports the analytical conclusion that radiosonde-sized payloads can have low and highly variable RCSs. The extracted cross-polarized RCS values in Figure 5 and Table 5 range from approximately −36.49 dBsm to −23.45 dBsm. This spread shows that radiosonde RCSs cannot be represented by a single fixed value. Detectability depends on orientation, polarization, and conductive internal parts, including sensors, the PCB, battery, and antenna. The EPS casing was omitted from the simplified FEM model; for this L-band case, that simplification is acceptable because these dielectric parts have only limited influence on the simulated response.

The 26 GHz M20 radiosonde simulation is used only as supplementary comparison data for the mm wave discussion. The updated Table 6 values range from −22.29 dBsm to +2.64 dBsm, indicating strong aspect dependence at the same nominal frequency. Because the radiosonde values in Table 6 are cross-polarized FEM results, they should not be compared directly with all DJI Matrice 100 polarization channels in Table 7. If the illustrative scaling from Equation (7) is applied to these 26 GHz M20 values, the corresponding 1.5 GHz range is approximately −47.07 to −22.14 dBsm for f^2^ scaling and −71.84 to −46.91 dBsm for f^4^ scaling. These scaled values are not measured L-band signatures. They only show the sensitivity of the comparison to aspect angle, polarization, target construction, and the selected frequency-scaling law.

To complement the calculations and simulations, Semkin et al. report measured mean RCS values for representative drones in the 26–40 GHz band. These data provide useful empirical context and show strong polarization dependence. The present study focuses on cross-polarized channels, although the co-polar channels (HH/VV) are consistently higher than the cross-polar channels (HV/VH). The lower and more irregular cross-polar signatures are consistent with shifting scattering centers across frequencies and polarization. This also shows that mean σ is not necessarily monotonic with frequency for complex airframes [33].

For illustration, the 26–40 GHz UAV mean RCS values were back-scaled to 1.5 GHz using Equation (7). In Table 8, the f^2^-scaled estimates for the DJI Matrice 100 cases range from −34.62 dBsm to −42.58 dBsm, while the f^4^ sensitivity case gives lower values between −59.56 dBsm and −70.64 dBsm. These values support the expectation of low L-band RCSs, but they should not be treated as definitive L-band target signatures. Operationally, such RCS levels suggest that L-band detection and tracking of C0–C2 UAVs and radiosonde-like objects may be intermittent and scenario-dependent. Performance in these cases usually depends strongly on geometry, clutter, processing, and integration conditions, not on nominal radar range alone.

### 4.2. Analyzing the Possible Detection Range of RAT-31DL as an L-Band Radar Example

The radar horizon was evaluated with the effective Earth-radius model, where the physical Earth radius RE is modified by the k = 4/3 effective Earth-radius factor to represent standard atmospheric refraction. For a 30 m radar antenna elevation, representative of a typical RAT-31DL installation, the calculated radar horizon for 60–140 m low-altitude targets is approximately 55–71 km. These values give the maximum geometric line-of-sight distance at which a target can theoretically be observed, provided that the radar beam also intersects the relevant altitude.

The RAT-31DL is an L-band 3D AESA radar. Public Leonardo material describes it as an L-band system with an effective range of more than 470 km for its general surveillance mission and mentions upgrades aimed at improving low-RCS target detection [38]. These figures should be interpreted cautiously, since detailed performance curves are not public and are strongly conditional. For very small RCS targets, detection can become highly scenario-dependent and may shift from stable tracking to intermittent or fragile tracks depending on geometry and clutter.

Antenna-elevation beam geometry shows that, in practical cases, low-altitude detectability can be limited before the radar horizon is reached. When the main beam is steered to a positive elevation angle, the beam axis rises with range and intersects a given low target altitude only up to a finite distance. Beyond that point, the target leaves the main-beam coverage regardless of radar power, sensitivity, or signal-processing capability.

Using a simplified beam-target intersection model, beam-axis intersection ranges were calculated for 60–140 m target altitudes and representative positive elevation angles between 2.5° and 20°. At 2.5° elevation, the main-beam axis intersects the examined low-altitude targets only out to approximately 0.69–2.52 km, and at 20°, to approximately 0.08–0.30 km. These distances are far smaller than the corresponding radar-horizon distances and show the dominant influence of elevation beam geometry in low-altitude detection.

This limitation is, quite literally, geometric. Although the RAT-31DL uses electronically steered elevation beams and advanced digital signal processing, those capabilities cannot change the fact that a beam steered above the horizontal will overshoot low-altitude targets as range increases. For low-altitude surveillance, the effective detection envelope is therefore determined not only by nominal radar range but also by the chosen elevation-beam strategy.

At system level, the results point to two geometric detection regimes. Near-horizontal beams are limited mainly by the radar horizon caused by Earth curvature, while positive-elevation beams are limited by beam-target intersection geometry at much shorter ranges. This difference is important when interpreting the operational strengths and limits of long-range surveillance radars.

RAT-31DL remains useful for LSS-related surveillance when its role is defined properly. It is best understood as a strategic L-band backbone for early warning, wide-area monitoring, and can provide tracking support of geometrically visible UAVs and UFBs with sufficient RCSs. Its limitations against very low-altitude, small, slow targets do not remove its value; they show where local sensors are needed. In a layered architecture, RAT-31DL provides wide-area surveillance, while tactical MMR and dedicated C-UAS radars improve local gap filling, tracking, classification, and low-altitude clutter-limited coverage. Table 12 summarizes this comparison.

### 4.3. Limitations of This Study

The analysis is limited in scope. First, it treats the RAT-31DL as a representative long-range, L-band 3D surveillance radar, so the results should not be generalized directly to all radar types with different waveforms, beam strategies, or processing chains. Second, only one nominal L-band operating point is considered, although small-target detectability and RCS can vary substantially with frequency and aspect angle. Third, the results rely on simplified analytical calculations and literature-based inputs rather than proprietary performance data or original field measurements. The FEM simulation also used a simplified radiosonde model in which the EPS casing and very small, mounted components were omitted. For the investigated L-band frequency range, this simplification is considered acceptable. Despite these limits, however, the study is useful because it identifies the main factors governing low-altitude performance for small RCS targets: geometric line-of-sight limits and elevation-beam geometry.

The radar-equation, CFAR, and SCR analyses are parametric because proprietary RAT-31DL waveform, PRF, integration, receiver-noise, loss, Doppler-filter, clutter-map, and threshold settings are not public. The radar-horizon calculation is also a smooth-Earth baseline; operational coverage prediction would require site-specific terrain, land cover, obstacle, and refractivity data. These limitations are stated explicitly to avoid interpreting the results as validated operational detection probabilities.

## 5. Conclusions

These RCS levels suggest that L-band detection and tracking of C0–C2 UAVs and UFB payloads, especially radiosonde-sized objects, may be intermittent and scenario-dependent. Because this study does not calculate probability of detection, it supports only a conditional detection assessment rather than a definitive detection claim.

The novelty of this work is that it brings RCS, radar horizon, beam geometry, clutter, and low-Doppler effects into one assessment. This makes the RAT-31DL result more realistic. It shows where the radar can support LSS detection, and where gap-filler sensors are still needed.

The parametric radar-equation section provides normalized, scenario-dependent detectability estimates rather than operational probability-of-detection claims. It identifies the main limiting mechanisms, which comprise RCS variability, geometric visibility, elevation-beam intersection, clutter, low-Doppler filtering, and tracker behavior.

Under these conditions, LSS detection depends mainly on geometric visibility, ground clutter, processing choices, and integration settings, not on nominal instrumented range alone. For UFB-related detections, response procedures should also account for legitimate civil and research balloon operations. Where applicable, established UFB classification schemes and standard safety measures, including airspace coordination, separation, and fail-safe design, can support risk-based assessment. This can reduce unnecessary alerts while maintaining protection against genuinely hostile LSS objects.

The results also show that the RAT-31DL, despite its long-range surveillance capability, cannot by itself provide continuous low-altitude coverage. Two geometric limitations dominate. With near-horizontal beams, coverage is bounded mainly by the radar horizon. With positive-elevation beams, low-altitude coverage quickly becomes beam-intersection-limited at ranges that may be orders of magnitude shorter than the horizon distance. These constraints are physical and cannot be removed by signal processing alone, although low-Doppler-preserving processing and suitable tracker logic are needed to avoid suppressing slow, clutter-adjacent targets.

RAT-31DL should therefore be treated as the strategic surveillance layer within a wider LSS architecture. Robust low-altitude UAV and UFB detection requires gap-filler sensors, low-Doppler-preserving processing, clutter-aware detection and tracker logic, terrain-aware deployment planning, and multi-sensor fusion.

## Figures and Tables

**Table 1:** Radar frequency range and wavelength range with band designation [9,10].

Nominal Frequency Range (GHz)	Wavelength Range (cm)	Band Designation
0.003–0.03	1000–10,000	HF
0.03–0.3	100–1000	VHF
0.3–1	30–100	UHF
1–2	15–30	L
2–4	7.5–15	S
4–8	3.7–7.5	C
8–12	2.5–3.7	X
12–18	1.7–2.5	K_u_
18–27	1.1–1.7	K
27–40	0.75–1.1	K_a_
40–75	0.4–0.75	V
75–110	0.27–0.4	W
110–300	0.1–0.27	mm
300–3000	0.01–0.1	THz

**Table 2:** Main detection and classification differences between small UAVs and radiosonde-type LUFB targets, synthesized from LSS, radar micro-Doppler, UFB operation, meteorological balloon, and C-UAS sensor fusion literature [19].

Feature	Small UAV	Radiosonde-Type LUFB Target
Motion pattern	Powered flight with maneuvering and hovering capability	Wind-drift dominated ascent, descent, or float
Doppler behavior	Body Doppler plus possible rotor micro-Doppler	Very low Doppler; no rotor-induced micro-Doppler
Dominant detection challenge	Low altitude, low RCS, clutter coupling, and aspect variability	Weak payload RCS, near-zero Doppler, and unclear drift-like track behavior
Main classification indicators	Rotor micro-Doppler, maneuvering, hover behavior, and track dynamics	Ascent/descent profile, wind-correlated drift, and correlation with known balloon operations
Preferred mitigation	C-UAS radar, passive RF, EO/IR acoustic sensing, and sensor fusion	Track history analysis, airspace coordination, low-Doppler preservation, and sensor fusion

**Table 3:** Calculated RCSs for basic radiosonde materials at 1.5 GHz; 26 GHz values are auxiliary references for later mm wave comparison [30,31].

Case	$\varepsilon_{r}$	$|\Gamma {|}^{2}$	σ @1.5 GHz (dBsm)	σ @26 GHz (dBsm)	Model
PEC reference plate (a)	-	1.00	−27.06	−2.29	$\sigma =\frac{4\pi A^{2}}{\lambda^{2}}$
PEC reference plate (b)	-	1.00	−15.02	9.76	$\sigma =\frac{4\pi A^{2}}{\lambda^{2}}$
EPS (a)	1.03	5.46 · 10^−5^	−69.69	−44.91	$\sigma_{dielectric}=\sigma_{PEC}\cdot |\Gamma {|}^{2}$
EPS (b)	1.03	5.46 · 10^−5^	−57.65	−32.87	$\sigma_{dielectric}=\sigma_{PEC}\cdot |\Gamma {|}^{2}$
FR-4 (a)	4.30	1.22 · 10^−1^	−36.20	−11.42	$\sigma_{dielectric}=\sigma_{PEC}\cdot |\Gamma {|}^{2}$
FR-4 (b)	4.30	1.22 · 10^−1^	−24.16	−0.62	$\sigma_{dielectric}=\sigma_{PEC}\cdot |\Gamma {|}^{2}$

**Table 4:** Electromagnetic material parameters used in the simplified FEM model at 1.5 GHz and 26 GHz [32].

Modeled Part	Assigned Material	Relative Permittivity ε_r_	Bulk Conductivity σ_e_ (S/m)	Skin Depth at 1.5 GHz (µm)	Skin Depth at 26 GHz (µm)	Modelling Role
PCB metallization and sensors	Copper	1.0	5.8 · 10^7^	1.7	0.4	Represents dominant highly conductive scattering elements
Antenna and metallic small parts	Stainless steel, 304-type nominal value	1.0	1.1 · 10^6^	11	2.9	Represents secondary conductive elements with lower conductivity

**Table 5:** M20 radiosonde’s RCS signatures of its sides (planes) at 1.5 GHz.

Plane and Direction	σ_dBsm_ Cross-Polarized (dBsm)
XY front	−27.07
XY back	−29.96
XZ front	−32.63
XZ back	−36.49
YZ front	−26.79
YZ back	−23.45

**Table 6:** M20 radiosonde’s RCS signatures of its sides (planes) at 26 GHz.

Plane and Direction	σ_dBsm_ Cross-Polarized (dBsm)
XY front	−13.44
XY back	−14.56
XZ front	−22.29
XZ back	−18.37
YZ front	−2.04
YZ back	+2.64

**Table 7:** RCS signatures of drone models at 26 GHz and 40 GHz [33].

Polarization	σ_dBsm_ at 26 GHz (dBsm)	σ_dBsm_ at 40 GHz (dBsm)
HH	−10.5	−6.6
VV	−10.0	−6.1
VH	−17.2	−13.1
HV	−17.8	−13.6

HH: Horizontal transmit to Horizontal receive; VV: Vertical transmit to Vertical receive; HV: Horizontal transmit to Vertical receive—it is cross-polarization; VH: Vertical transmit to Horizontal receive—it is cross-polarization.

**Table 8:** Mean estimates for DJI Matrice 100’s RCS.

Polarization	σ_dBsm_ at 1.5 GHz from 26 GHz (dBsm) @ f^2^	σ_dBsm_ at 1.5 GHz from 26 GHz (dBsm) @ f^4^	σ_dBsm_ at 1.5 GHz from 40 GHz (dBsm) @ f^2^	σ_dBsm_ at 1.5 GHz from 40 GHz (dBsm) @ f^4^
HH	−35.28	−60.06	−35.12	−63.64
VV	−34.78	−59.56	−34.62	−63.14
VH	−41.98	−66.76	−41.62	−70.14
HV	−42.58	−67.36	−42.12	−70.64

**Table 9:** Calculated geometric visibility and beam-axis intersection ranges of the RAT-31DL radar according to different initial conditions.

Elevation Tilt Angle (°)	Target Altitude (m)	Geometric Visibility and Beam-Axis Intersection Range (km)
0	60	54.5
	80	59.4
	100	63.8
	120	67.7
	140	71.3
2.5	60	0.69
	80	1.15
	100	1.60
	120	2.06
	140	2.52
10	60	0.17
	80	0.28
	100	0.40
	120	0.51
	140	0.62
20	60	0.08
	80	0.14
	100	0.19
	120	0.25
	140	0.30

**Table 10:** Detection range sensitivity to small-target RCSs under fixed radar-equation parameters.

Target RCS (dBsm)	Linear σ (m^2^)	Relative Range Factor
−24	3.98 · 10^−3^	0.251
−27	2.00 · 10^−3^	0.211
−30	1.00 · 10^−3^	0.178
−33	5.01 · 10^−4^	0.150
−36	2.51 · 10^−4^	0.126

**Table 11:** Low-Doppler sensitivity of LSS targets at the L-band.

Radial Velocity v_r_ (m/s)	Doppler Shift at 1.5 GHz (Hz)
1	10
5	50
10	100
20	200
50	500
100	1000

**Table 12:** Horizontal comparison between RAT-31DL and complementary LSS-detection radar classes [39,40].

Radar/System Class	Typical Band	Primary Role	Relevance to LSS Detection
RAT-31DL	L-band	Long-range 3D air surveillance	Strategic coverage; low-altitude LSS performance constrained by horizon, clutter, beam geometry, and processing settings
X/Ku-band C-UAS radar	X/Ku or higher	Short-range LSS/UAV detection and classification	Higher angular/range resolution and stronger micro-Doppler exploitation, but shorter practical coverage
Multistatic/passive radar	Emitter-dependent	Networked detection and classification	Multiple viewing geometries can improve discrimination and reduce single-radar shadowing

## Data Availability

The original contributions presented in this study are included in the article. Further inquiries can be directed to the corresponding author.

## Abbreviations

The following abbreviations are used in this manuscript:

A	Area
A_c_	Illuminated Clutter-Cell Area
AESA	Active Electronically Scanned Array
AGL	Above Ground Level
AMTI	Adaptive Moving Target Indication
B	Receiver Bandwidth
c	Speed of Light
C0	UAV Class Mark in EU
C1	UAV Class Mark in EU
C2	UAV Class Mark in EU
CA-CFAR	Cell Averaging-Constant False Alarm Rate
CFAR	Constant False Alarm Rate
cm	Centimeter
CPI	Coherent Processing Interval
C-RAM	Counter Rocket Artillery and Mortar
C-UAS	Counter-Unmanned Aircraft System
dB	Decibel
dBsm	Decibels Relative to 1 m^2^
D_f_	Dissipation Factor
Dk	Dielectric Constant
d_km_	Radar Horizon Distance (km)
dm	Decimeter
DTI	Detection, Tracking and Identification
d_θ, km_	Last Beam-Target Intersection Distance
EASA	European Union Aviation Safety Agency
EO/IR	Electro-Optical / Infrared
EPS	Expanded Polystyrene
f_D_	Doppler Frequency Shift
FEM	Finite Element Method
F_n_	Receiver Noise Figure, Linear Factor
g	Gram
G	Monostatic Antenna Gain
GHz	Gigahertz
G_i_	Integration Gain / Processing Gain
GO-CFAR	Greatest Of-Constant False Alarm Rate
G_r_	Receive Antenna Gain
G_t_	Transmit Antenna Gain
h_c_	Target Altitude Above Ground Level
HH	Horizontal Transmit to Horizontal Receive
h_r_	Radar Antenna Elevation Above Ground Level
HV	Horizontal Transmit to Vertical Receive
Hz	Hertz
k	Effective Earth Radius Factor
k_B_	Boltzmann Constant
kg	Kilogram
kHz	Kilohertz
L	Total System Loss Factor
LDM	Low Doppler Map
LSS	Low, Slow, Small
LUFB	Light Unmanned Free Balloon
m	Meter
MHz	Megahertz
mm	Millimeter
MMR	Multi-Mission Radar
MTI	Moving Target Indication
MTOM	Maximum Takeoff Mass
NATINAMDS	NATO’s Integrated Air and Missile Defense System
OS-CFAR	Order Statistics-Constant False Alarm Rate
PCB	Printed Circuit Board
P_d_	Probability of Detection
PEC	Perfect Electric Conductor
P_fa_	Probability of False Alarm
P_n_	Receiver Noise Power
P_r_	Received Echo Power
PRF	Pulse Repetition Frequency
P_t_	Transmitted Power
R	Radar-to-Target Range
RCS	Radar Cross-Section
R_E_	Earth Radius
RF	Radio Frequency
R_max_	Maximum Detection Range
SCR	Signal-to-Clutter Ratio
SNR	Signal-to-Noise Ratio
SNR_min_	Minimum Required Signal-to-Noise Ratio
SO-CFAR	Smallest Of-Constant False Alarm Rate
T_0_	Reference Noise Temperature
THz	Terahertz
T_S_	System Noise Temperature
UAV	Unmanned Aerial Vehicle
UFB	Unmanned Free Balloon
VH	Vertical Transmit to Horizontal Receive
v_r_	Radial Velocity
VV	Vertical Transmit to Vertical Receive
XPS	Extruded Polystyrene
∝	Proportional
tanδ	Loss Tangent
Γ	Electric-Field Reflection Coefficient
|Γ|^2^	Power Reflectivity
γ0	Normalized Clutter Reflectivity
ε_r_	Relative Permittivity
θ	Elevation Tilt Angle
λ	Wavelength
μ_r_	Relative Permeability
ρ_e_	Electrical Resistivity
σ	Radar Cross-Section
σ_c_	Clutter-Equivalent Radar Cross-Section
σ_e_	Electrical Conductivity
σ_t_	Target Radar Cross-Section
